# Granulocyte colony‐stimulating factor may improve engraftment in adults with sickle cell disease undergoing non‐myeloablative haematopoietic stem cell transplantation

**DOI:** 10.1111/bjh.70093

**Published:** 2025-08-15

**Authors:** Shaoshi Zhu, Lucas Maahs, Karen Sweiss, Sally A. Campbell‐Lee, Nadim Mahmud, Victor R. Gordeuk, Damiano Rondelli, Santosh L. Saraf

**Affiliations:** ^1^ College of Medicine University of Illinois Chicago Chicago Illinois USA; ^2^ Division of Hematology & Oncology, Department of Medicine University of Illinois Chicago Chicago Illinois USA; ^3^ Department of Pharmacy Practice University of Illinois Chicago Chicago Illinois USA; ^4^ Department of Pathology University of Illinois Chicago Chicago Illinois USA

**Keywords:** engraftment, G‐CSF, haematopoietic stem cell transplantation, non‐myeloablative, sickle cell disease

To the Editor,

Sickle cell disease (SCD) is an inherited red blood cell disorder caused by a mutation in the β‐globin gene leading to the production of abnormal haemoglobin S. Despite advances in management, SCD still causes significant morbidity and shortened life expectancy.^1^ Allogeneic haematopoietic stem cell transplantation (HSCT) is a potentially curative therapy used primarily in patients with severe SCD‐related complications, such as frequent vaso‐occlusive episodes, acute chest syndrome or stroke. The majority of HSCT for SCD is performed in children using myeloablative‐conditioning regimens, but these approaches carry a greater risk of graft‐versus‐host disease and mortality in adults.^2^ Non‐myeloablative conditioning regimens, such as the alemtuzumab–total body irradiation regimen, with human leucocyte antigen (HLA)‐matched sibling donors have been increasingly utilized in adults with SCD.^3^ While this regimen is relatively well tolerated with low rates of acute or chronic graft‐versus‐host disease, there is a greater risk of mixed donor–recipient chimerism and graft loss with this conditioning approach.^3^, ^4^

Since July 2016, our centre has administered granulocyte colony‐stimulating factor (G‐CSF) in the post‐HSCT setting to SCD patients in an attempt to reduce the duration of neutropenia and potentially improve donor chimerism values. Here, we report on the safety and efficacy of G‐CSF in adults with SCD who underwent allogeneic HSCT using the alemtuzumab–total body irradiation‐conditioning regimen with HLA‐matched sibling donors between November 2011 and August 2021.

The study was approved by the University of Illinois Chicago Institutional Review Board and all patients provided written informed consent before enrolment (NCT01499888).

Patients received a red blood cell exchange transfusion on day −10 to achieve a haemoglobin S <30% followed by alemtuzumab (1 mg/kg total dose intravenous; days −7 to −3), total body irradiation (3 Gy; day −2) and sirolimus (starting day −1; trough goal 5–15 ng/mL). G‐CSF (5 mg/kg subcutaneous per day) was administered to all patients undergoing HSCT starting in July 2016 after day +5 post‐HSCT and after confirming haemoglobin S <30%. G‐CSF was administered only on days when the absolute neutrophil count (ANC) was <0.5 x 10^9^ neutrophils/L. We compared maximum pain scores, time to neutrophil engraftment and incidence of bacterial infections or neutropenic fever in those that received G‐CSF (*n* = 13) versus those that did not (*n* = 21). Neutrophil engraftment was defined as an ANC >0.5 x 10^9^ neutrophils/L for three consecutive days without requiring G‐CSF support. Patients remained inpatient until neutrophil engraftment and all doses of G‐CSF were administered in the hospital setting. Whole blood chimerism values were compared between groups at the respective time points. We compared lymphoid (CD3) and myeloid (CD33) chimerism values, which became routinely available at our centre in 2016, at the last assessment between those that received and did not receive G‐CSF post‐HSCT. Patients were tapered off immunosuppression once the CD3 chimerism values were >40% donor. Secondary graft failure was defined as whole blood or myeloid donor chimerism values <10%.^5^

Linear variables were compared by G‐CSF use with the Mann–Whitney or Student's *t*‐test. Categorical variables were compared by G‐CSF use with the chi‐squared or Fisher exact test. We compared the Kaplan–Meier curves for graft failure‐free survival using the log‐rank method. Median and interquartile range (IQR) values are provided.

Baseline characteristics of adults with SCD undergoing HSCT by G‐CSF status are summarized in Table 1. There was a trend for those receiving G‐CSF being older and more commonly male than those who did not receive G‐CSF; CD34+ cell dose, SCD genotype, donor–recipient major ABO mismatch and red blood cell alloimmunization patterns were similar between the two groups. For patients who received G‐CSF, the median day of G‐CSF initiation was day +10 post‐HSCT (IQR, day +7 to day +17) and the median duration of use was 3 days (IQR, 2–7 days). Four of the 13 patients treated with G‐CSF had a decline in the ANC <500 neutrophils/μL after the G‐CSF was stopped and had the G‐CSF restarted.

**TABLE 1:** Characteristics of patients with sickle cell disease undergoing haematopoietic stem cell transplantation with versus without G‐CSF support.

	No G‐CSF (*n* = 21)	G‐CSF (*n* = 13)	*p*‐Value
Age	32 (25–38)	37 (30–51)	0.07
Male:female	57%:43%	69%:31%	0.1
Haemoglobin SS	19 (90%)	11 (85%)	0.1
Major ABO mismatch	4 (19%)	1 (8%)	0.6
Minor ABO mismatch	3 (14%)	3 (23%)	0.7
Recipient alloimmunization	5 (24%)	3 (23%)	1.0
Alloantibody against non‐ABO donor RBC antigens	1 (5%)	1 (8%)	1.0
CD34+ dose (×10^6^/kg)	8.1 (5.8–9.8)	8.6 (7.4–9.2)	0.5
Maximum pain score during hospitalization (out of 10)	10 (9–10)	8 (7–10)	0.08
Neutrophil engraftment (days)	22 (12–29)	16 (12–23)	0.2
Days neutropenic	9 (0–15)	7 (3–11)	0.9
Neutropenic fever (%)	6 (29%)	1 (8%)	0.1
Bacterial infection (%)	10 (48%)	4 (31%)	0.3
Secondary graft loss (%)	5 (24%)	0 (0%)	0.06

*Note*: Median and interquartile range values are provided.

Abbreviations: G‐CSF, granulocyte colony‐stimulating factor; RBC, red blood cell.

We did not observe a vaso‐occlusive episode or acute chest syndrome event during the hospitalization for HSCT in either SCD patients receiving or not receiving G‐CSF in the post‐HSCT period. The maximum pain score that was recorded during the hospitalization for HSCT was similar between the two groups (Table 1). Neutrophil engraftment and the duration of neutropenia were also similar between the two groups. The incidence of neutropenic fever and bacterial infections was lower in the G‐CSF group than in the patients who did not receive G‐CSF, but the differences were not statistically significant. Whole blood chimerism values were significantly higher in the SCD patients who received versus did not receive G‐CSF at all time points up to 2‐year post‐HSCT (Figure 1A). Myeloid chimerism values were significantly higher and lymphoid chimerism values trended higher in SCD patients who received versus did not receive G‐CSF post‐HSCT (Figure 1B). Furthermore, we observed no secondary graft loss in the G‐CSF group as compared to five patients with secondary graft loss in the no G‐CSF group (Table 1; Figure 1C). Among patients with stable engraftment, there was a trend towards more patients who received G‐CSF being successfully tapered off immunosuppression (11/13, 85%) versus patients who did not receive G‐CSF post‐HSCT (10/16, 63%) (*p* = 0.19).

**FIGURE 1:**
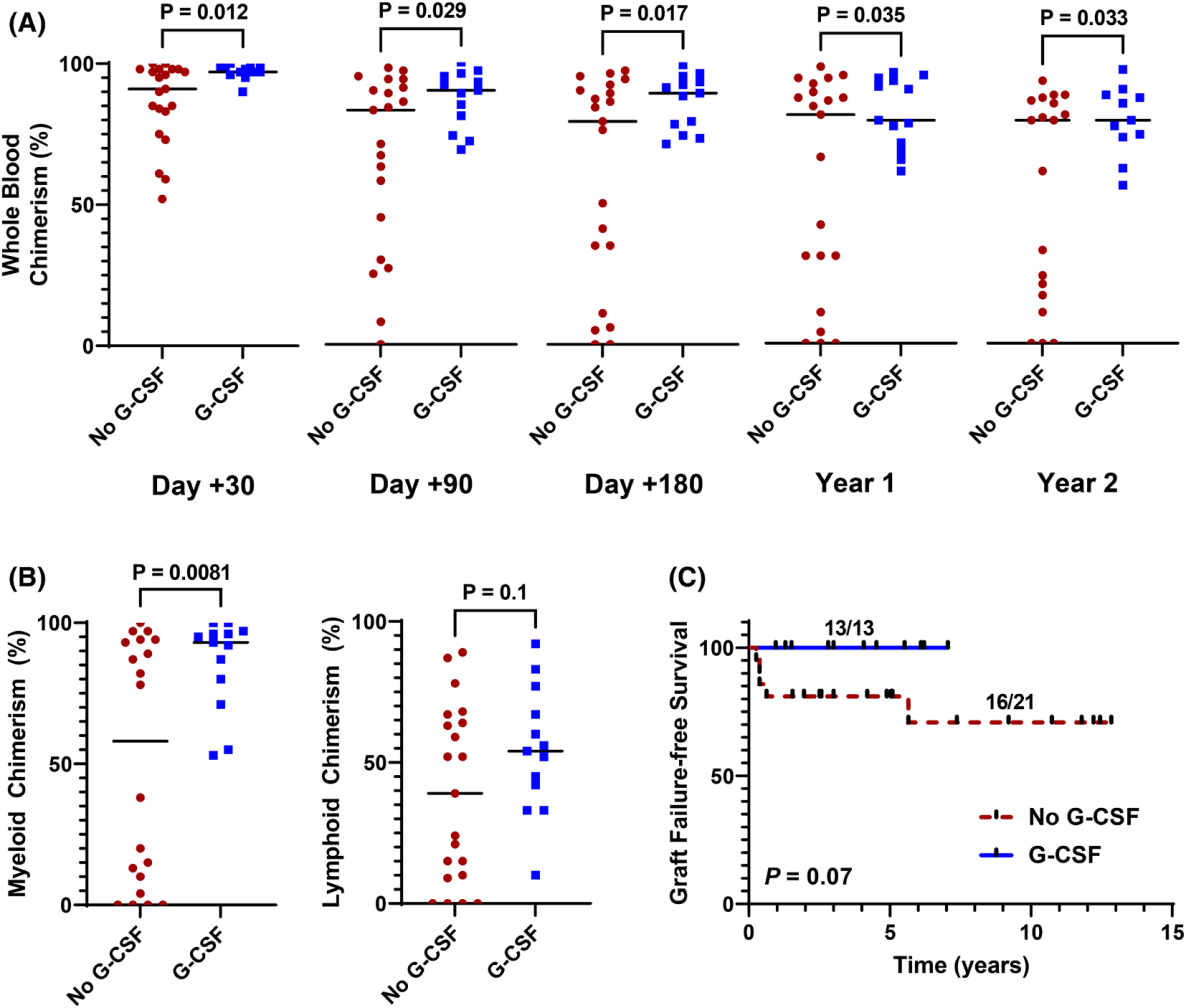
(A) Whole blood donor chimerism values post‐HSCT. Whole blood chimerism values were significantly higher in SCD patients who received G‐CSF versus those who did not at day +30 (G‐CSF: 97% [97%–99%]; no G‐CSF 91% [81%–98%]), day +90 (G‐CSF: 90% [79%–95%]; no G‐CSF 83% [41%–92%]), day +180 (G‐CSF: 89% [77%–94%]; no G‐CSF 79% [29%–91%]), 1 year (G‐CSF: 79% [70%–93%]; no G‐CSF 81% [26%–90%]) and 2 years (G‐CSF: 79% [73%–88%]; no G‐CSF: 79% [18%–86%] post‐HSCT). Median and [interquartile range] values are provided. (B) Myeloid and lymphoid chimerism values at the last available assessment by G‐CSF status post‐HSCT. The most recent myeloid and lymphoid donor chimerism values were higher in those that received G‐CSF (myeloid: 93% [78%–96%]; lymphoid: 54% [40%–70%]; median time of assessment post‐HSCT 4.5 [2.5–6.2] years) than in those who did not receive G‐CSF (myeloid: 58% [6%–94%]; lymphoid: 39% [10%–65%]; median time of assessment post‐HSCT 4.9 [1.9–11.1] years) post‐HSCT. Median and [interquartile range] values are provided. (C) Graft failure‐free survival in patients by G‐CSF status post‐HSCT. G‐CSF, granulocyte colony‐stimulating factor; HSCT, haematopoietic stem cell transplantation; SCD, Sickle cell disease.

In this study, we demonstrate that administrating G‐CSF to adults with SCD undergoing HSCT using the alemtuzumab–total body irradiation approach is well tolerated once the HbS is lowered to <30% by red blood cell exchange transfusion and associated with higher whole blood and myeloid donor chimerism values. There were also trends for higher lymphoid chimerism values, a greater proportion of patients discontinuing immunosuppression and lower rates of secondary graft loss in those who received versus did not receive G‐CSF post‐HSCT.

Our study builds upon findings demonstrating the safety of G‐CSF in patients with SCD in the post‐HSCT setting. There have been reports of SCD‐related complications, such as severe vaso‐occlusive episodes, acute chest syndrome or multi‐organ system failure, when G‐CSF has been administered to patients with SCD in the non‐HSCT setting.^6^ In this case series, patients who received an exchange red blood cell transfusion with a goal haemoglobin S <30% did not experience SCD‐related complications with G‐CSF use.^6^ In a cohort of 62 children and young adults with a median age of 10 years, G‐CSF administered after a fludarabine–melphalan conditioning regimen and ensuring that the haemoglobin S <45% was well tolerated without an increased risk of SCD‐related complications.^7^ Our findings demonstrate that G‐CSF is also well tolerated in adults with SCD in the post‐HSCT setting. We did not observe any SCD‐related complications, such as a vaso‐occlusive episode or acute chest syndrome, nor did pain scores worsen with G‐CSF use after confirming haemoglobin S <30%. Together, these findings support the judicious use of G‐CSF in patients with SCD after ensuring that the haemoglobin S% has been lowered with red blood cell transfusion therapy, preferably to a goal of <30%.

We also observed that G‐CSF administered in the post‐HSCT setting is associated with higher donor chimerism values and a trend for less secondary graft loss. One potential explanation for this observation is based on the ability of G‐CSF to increase the expression of high‐affinity IgG receptors on neutrophils thereby enhancing antibody‐dependent cellular cytotoxicity.^8^ In one study, G‐CSF exposure increased neutrophil‐mediated antithymocyte globulin cytotoxicity by 40‐fold, leading to increased T‐cell clearance in the post‐HSCT setting.^9^ Our conditioning regimen uses alemtuzumab, another in vivo T‐cell depleting agent. Our observation of higher donor chimerism values and stable engraftment with G‐CSF use post‐HSCT may be due to enhanced T‐cell clearance. Alternative explanations for the higher donor chimerism values with G‐CSF use could include differing effects on myeloid progenitor cells when administered on day +5 post‐HSCT or later^10^ or alteration of endosteal cytokines or niche‐supportive macrophages and osteoblasts in the bone marrow microenvironment favouring the donor stem cells.^11^ These potential effects should be evaluated in future studies.

The alemtuzumab–total body irradiation HSCT regimen has helped expand access for curative therapies to adults with SCD, although the need for long‐term immunosuppression and secondary graft loss in some patients may prevent its widespread use.^3^, ^4^ While our study is limited by the small sample size and being from a single centre, we demonstrate encouraging results that G‐CSF administered post‐HSCT may help mitigate the risk of secondary graft loss with this non‐myeloablative approach. Although both groups had a similar median time of assessment for last chimerism values, in part due to four of the subjects in the no‐G‐CSF group having secondary graft loss within a year post‐HSCT, long‐term follow‐up will be important to determine whether the stable engraftment with G‐CSF persists over time. Another limitation of our study is that we are unclear whether patients who have remained on immunosuppression >2 years for low lymphoid chimerism values require long‐term immunosuppression. Future studies investigating the effects of G‐CSF on T‐cell clearance and donor myeloid progenitor cells may guide strategies to improve outcomes for curative therapies in individuals with SCD.

## AUTHOR CONTRIBUTIONS

Study conception and design: SZ, LM, KS, SAC‐L, NM, VRG, DR, SLS. Data collection, analysis and interpretation of results: SZ, LM, KS, SAC‐L, NM, VRG, DR, SLS. Draft manuscript preparation: SZ, LM, KS, SACL, NM, VRG, DR, SLS. All authors critically reviewed and approved the final version of this manuscript.

## CONFLICT OF INTEREST STATEMENT

VRG: No relevant COI related to this manuscript but has served as a consultant for Agios, Global Blood Therapeutics/Pfizer, GlaxoSmithKline and Vertex Pharmaceuticals and received research funding from Incyte and Novartis. SLS: No relevant COI related to this manuscript but has served on advisory boards or as a consultant for Agios, BEAM therapeutics, Forma/Novo Nordisk, Novartis, Global Blood Therapeutics/Pfizer, Chiesi and Fulcrum. SZ, LM, KS, SAC‐L, NM and DR have no relevant COI to report.

## Data Availability

The data that support the findings of this study are available upon reasonable request from the corresponding author.
